# Mixed Incoherent Far-Field and Near-Field Source Localization under Uniform Circular Array

**DOI:** 10.3390/s18051432

**Published:** 2018-05-04

**Authors:** Xiaolong Su, Zhen Liu, Xin Chen, Xiang Li

**Affiliations:** College of Electronic Science, National University of Defense Technology, Changsha 410073, China; suxiaolong_nudt@163.com (X.S.); chenxin10@nudt.edu.cn (X.C.); lixiang01@vip.sina.com (X.L.)

**Keywords:** mixed sources, uniform circular array (UCA), least squares method, parameter estimation, phase difference

## Abstract

A high-accuracy algorithm is presented for the localization of mixed incoherent near-field and far-field narrow-band sources under uniform circular array (UCA). Herein, considering that it is difficult to classify the mixed sources, we first decouple mixed sources’ angles and ranges by calculating centro-symmetric sensors’ phase differences. Then, as the phase differences including only sources’ angles can be transformed as indefinite equations, each source’s azimuth angle and elevation angle are obtained by performing the least squares method. After that, on the basis of the estimated angles of the mixed sources, one-dimensional (1-D) multiple signal classification (MUSIC) method and corresponding spatial spectrum are utilized to identify the mixed sources and estimate the ranges of the near-field sources. Finally, simulation and comparison results verify the superior performance of our proposed algorithm.

## 1. Introduction

Passive source localization using an array of spatially separated sensors is significant in the field of sonar, radar, wireless communication, etc. Many super-resolution algorithms have been presented to solve the localization of pure near-field sources (NFSs) condition or pure far-field sources (FFSs) condition [1,2,3,4,5,6]. However, considering some practical applications, such as speaker localization via microphone array and guidance system, speakers may be in the mixed near-field or far-field of the array location. Due to the fact that most super-resolution algorithms inherently require the radiation sources to be pure near-field sources or pure far-field sources, traditional methods may fail in such mixed circumstances [7,8,9,10,11,12,13].

Considering the configuration of the symmetric uniform linear array (ULA) and the mixed source condition, spatial differencing technique is performed to identify the near-field sources from the mixed sources and obtain the elevation angles and ranges of the near-field sources along with the elevation angles of the far-field sources, respectively [12]. However, the aforementioned method has limitation because the configuration of ULA can only be used in the condition of two-dimensional (2-D) localization (elevation angle and range). The array structure of the uniform circular array (UCA) has all-round azimuthal coverage and additional elevation angle information, which is preferable over the array structure of ULA [2,3,4,5]. As for the localization of 3-D condition (azimuth angle, elevation angle and range), based on the centro-symmetric configuration of UCA, Wu et al. [4] employed 2-D multiple signal classification (MUSIC) method to search the 2-D direction of arrivals (DOAs), and further utilized 1-D MUSIC method to estimate corresponding ranges, which can handle multiple near-field sources. Employing the phase of the adjacent sensors’ correlation function, Chen et al. [14] presented a phase-based algorithm to estimate the 2-D DOAs and ranges of multiple near-field sources, which is more computationally simple. However, due to the fact that it is difficult to classify mixed sources, the aforementioned methods cannot be extended to the localization for mixed near-field and far-field sources.

Recently, by employing differencing matrix, Xue et al. [13] first employed differencing matrix to separate the near-field sources from the mixed sources and obtain the 2-D DOAs of the near-field sources. Then, 1-D MUSIC method and 2-D MUSIC method are utilized to obtain the ranges of near-field sources and the 2-D DOAs of far-field sources, respectively. However, this method shows an unsatisfactory performance because it needs an expensive search process and thus suffers from computational cost. Therefore, the goal of this paper is to explore how to utilize the efficient methodology such as the phase-based algorithm for the mixed source localization under UCA.

In this paper, according to the centro-symmetric property of a fixed UCA, we presents a closed-form algorithm which extends the scheme in [3] to estimate the 2-D DOAs of mixed near-field and far-field narrow-band sources. Firstly, considering that it is difficult to classify mixed sources, we decouple angle parameters from array direction vector by calculating centro-symmetric sensors’ phase differences. After that, as the phase differences can be expressed as indefinite equations, we perform the least squares method to estimate the 2-D DOAs of the mixed sources. Meanwhile, substituting the obtained 2-D DOAs into the steering vector of the near-field source, this paper employs 1-D MUSIC method to obtain the ranges of near-field sources. Moreover, according to different convergence of near-field source’s range and far-field source’s range, we utilize the range spatial spectrum to identify the mixed sources. Compared with the two-stage MUSIC (TSMUSIC) approach [13], the proposed algorithm is computationally simple and provides the 2-D DOAs of all sources simultaneously. Simulation results verify that the proposed algorithm can realize mixed incoherent source localization with satisfactory performance.

## 2. Signal Model

**Figure 1:**
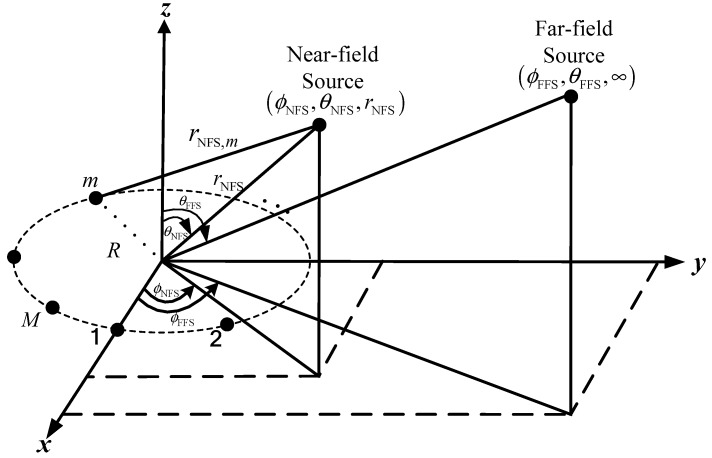
Geometry of a UCA with mixed near-field and far-field sources.

A UCA with radius R and M identical omnidirectional sensors impinged by P incoherent narrow-band signals is considered in this paper, its geometry is shown in Figure 1. The mixed narrow-band sources contains $P_{\mathrm{NFS}}$ near-field sources and $P_{\mathrm{FFS}}$ far-field sources. Each source has a different frequency. The sensors are uniformly distributed on the circumference and the first sensor is located at the *x*-axis. Moreover, phase reference point is the array center and all sensors are employed on a plane. The near-field source comes from $\left(\phi_{\mathrm{NFS}},\;\theta_{\mathrm{NFS}},\;r_{\mathrm{NFS}}\right)$ and the far-field source comes from $\left(\phi_{\mathrm{FFS}},\theta_{\mathrm{FFS}},\infty \right)$, where ϕ∈[0,2π) is the azimuth angle which is measured counterclockwise from the *x*-axis, θ∈[0,π/2) is the elevation angle which is measured downward from the *z*-axis and r is the range which is measured from the array center.

The received data of the *m*th sensor in the UCA at the *n*th sample can be expressed as

(1) $$x_{m}^{}\left[n\right]=\sum_{p=1}^{P}s_{p}\left[n-\tau_{m}\right]+w_{m}^{}\left[n\right]$$

for m=1,2,…,M and n=1,2,…,N, where $s_{p}\left[n\right]$ denotes the *p*th narrow-band source sampling with power $\sigma_{s,p}^{2}$, $w_{m}^{}\left[n\right]$ is independent of s[n], which is assumed to be a zero-mean white complex Gaussian noise with power $\sigma_{n}^{2}$, $\tau_{m}$ is the propagation delay of the *m*th sensor when taking array center as a benchmark, and the propagation delay of far-field source can be expressed as

(2) $$\tau_{\mathrm{FFS},m}=R\cos(\gamma_{m}-\phi_{\mathrm{FFS}})\sin\theta_{\mathrm{FFS}}/c$$

where $\gamma_{m}=2\pi (m-1)/M$ and c is the speed of light. Considering that near-field source is in the Fresnel region of the array aperture, the wavefront have to be characterized by azimuth angle, elevation angle and range. Therefore, taking the array center as a benchmark, the propagation delay of near-field source for the *m*th sensor can be expressed as

(3) $$\tau_{\mathrm{NFS},m}=(r_{\mathrm{NFS},m}(\phi_{\mathrm{NFS}},\theta_{\mathrm{NFS}},r_{\mathrm{NFS}})-r_{\mathrm{NFS}})/c$$

where $r_{\mathrm{NFS},m}(\phi_{\mathrm{NFS}},\theta_{\mathrm{NFS}},r_{\mathrm{NFS}})$ is the range between the near-field source and the *m*th sensor, which can be given by

(4) $$r_{\mathrm{NFS},m}(\phi_{\mathrm{NFS}},\theta_{\mathrm{NFS}},r_{\mathrm{NFS}})=\sqrt{{r_{\mathrm{NFS}}}^{2}+R^{2}-2r_{\mathrm{NFS}}R\zeta_{m}(\phi_{\mathrm{NFS}},\theta_{\mathrm{NFS}})}$$

where $\zeta_{m}(\phi_{\mathrm{NFS}},\theta_{\mathrm{NFS}})=\cos(\gamma_{m}-\phi_{\mathrm{NFS}})\sin\theta_{\mathrm{NFS}}$ with $\gamma_{m}=2\pi (m-1)/M$. Considering a second-order Taylor series expansion around the point where the value of $R/r_{\mathrm{NFS}}$ is approximated to zero, $r_{\mathrm{NFS}}{}_{,m}(\phi_{\mathrm{NFS}},\theta_{\mathrm{NFS}},r_{\mathrm{NFS}})$ can be well simplified as

(5) $$r_{\mathrm{NFS},m}(\phi_{\mathrm{NFS}},\theta_{\mathrm{NFS}},r_{\mathrm{NFS}})\approx r_{\mathrm{NFS}}-R\zeta_{m}(\phi_{\mathrm{NFS}},\theta_{\mathrm{NFS}})+\left(R^{2}/2r_{\mathrm{NFS}}\right)\left(1-\zeta_{m}^{2}(\phi_{\mathrm{NFS}},\theta_{\mathrm{NFS}})\right)$$

Because the difference in the envelope of the narrow-band signal can be negligible on each sensor, substituting (2), (3) and (5) into (1) obtains the mixed incoherent narrow-band near-field and far-field signal model

(6) $$\begin{array}{ll} x_{m}\left[n\right]= & \sum_{p=1}^{P_{\mathrm{NFS}}}s_{p}\left[n\right]\exp\left\{\frac{j2\pi Rf_{p}}{c}\left[\zeta_{m}(\phi_{p},\theta_{p})-\frac{R}{2r_{p}}\left(1-\zeta_{m}^{2}(\phi_{p},\theta_{p})\right)\right]\right\}\\ & +\sum_{p=P_{\mathrm{NFS}}+1}^{P_{\mathrm{NFS}}+P_{\mathrm{FFS}}}s_{p}\left[n\right]\exp\left\{\frac{j2\pi Rf_{p}}{c}\zeta_{m}(\phi_{p},\theta_{p})\right\}+w_{m}\left[n\right] \end{array}$$

## 3. Proposed Algorithm

It can be noticed that exponent part in (6) includes the azimuth angle, elevation angle and range parameters of the mixed sources. As the incoherent sources can be resolved in frequency domain, this paper first utilizes frequency spectrum to distinguish mixed incoherent sources and find the corresponding phase of each spectrum peak in phase spectrum. Then, extending the scheme in [3], we decouple and obtain the 2-D DOAs of every single source by calculating the phase difference of the centro-symmetric sensors. Finally, on the basis of the estimated angles of mixed sources, 1-D MUSIC method is utilized to classify mixed sources and obtain the ranges of near-field sources.

### 3.1. 2-D DOAs Estimation

When the number of sensors under UCA is even, it can be seen that $\gamma_{m+M/2}=\gamma_{m}+\pi$ and $\zeta_{m+M/2}(\phi ,\theta )=-\zeta_{m}(\phi ,\theta )$. Consider the correlation function of the *m*th centro-symmetric sensors for a single near-field source expressed as

(7) $$\begin{array}{ll} R_{\mathrm{NFS},m}=E\left[x_{\mathrm{NFS},m}x_{\mathrm{NFS},m+M/2}^{*}\right] & =\sigma_{s,\mathrm{NFS}}^{2}a_{\mathrm{NFS},m}a_{\mathrm{NFS},m+M/2}^{*}+\sigma_{n,\mathrm{NFS}}^{2}\\ & =\sigma_{s,\mathrm{NFS}}^{2}\exp\left\{\left(j4\pi Rf/c\right)\cos\left(\gamma_{m}-\phi_{\mathrm{NFS}}\right)\sin\theta_{\mathrm{NFS}}\right\}+\sigma_{n,\mathrm{NFS}}^{2} \end{array}$$

and the correlation function for a single far-field source expressed as

(8) $$\begin{array}{ll} R_{\mathrm{FFS},m}=E\left[x_{\mathrm{FFS},m}x_{\mathrm{FFS},m+M/2}^{*}\right] & =\sigma_{s,\mathrm{FFS}}^{2}a_{\mathrm{FFS},m}a_{\mathrm{FFS},m+M/2}^{*}+\sigma_{n,\mathrm{FFS}}^{2}\\ & =\sigma_{s,\mathrm{FFS}}^{2}\exp\left\{\left(j4\pi Rf/c\right)\cos\left(\gamma_{m}-\phi_{\mathrm{FFS}}\right)\sin\theta_{\mathrm{FFS}}\right\}+\sigma_{n,\mathrm{FFS}}^{2} \end{array}$$

for m=1,2,…,M/2 and (•)* denotes the complex conjugation, where $x_{\mathrm{NFS},m}\in {\mathbb{C}}^{1\times N}$ and $x_{\mathrm{FFS},m}\in {\mathbb{C}}^{1\times N}$ respectively represents the sampling data of the single near-field source and the single far-field source in the *m*th sensor, $\sigma_{s,\mathrm{NFS}}^{2}$ and $\sigma_{s,\mathrm{FFS}}^{2}$ respectively represents the power of the single near-field source and the single far-field source, $a_{\mathrm{NFS},m}$ and $a_{\mathrm{FFS},m}$ respectively represent the direction parameters of the near-field source and the far-field source at the *m*th sensor, which can be expressed as

(9) $$a_{\mathrm{NFS},m}=\exp\left\{\left(j2\pi Rf/c\right)\left[\zeta_{m}(\phi_{\mathrm{NFS}},\theta_{\mathrm{NFS}})-\left(R/2r_{\mathrm{NFS}}\right)\left(1-\zeta_{m}^{2}(\phi_{\mathrm{NFS}},\theta_{\mathrm{NFS}})\right)\right]\right\}$$

(10) $$a_{\mathrm{FFS},m}=\exp\left\{\left(j2\pi Rf/c\right)\zeta_{m}(\phi_{\mathrm{FFS}},\theta_{\mathrm{FFS}})\right\}$$

Assuming the received data is noiseless, it can be noticed that the phase of $R_{\mathrm{NFS},m}$ and $R_{\mathrm{FFS},m}$ can be unified as

(11) $$u_{m}=(4\pi Rf/c)\cos(\gamma_{m}-\phi )\sin\theta$$

Accordingly, as the phase of correlation function represents the phase difference of centro-symmetric sensors and the incoherent sources can be resolved in frequency domain, this paper utilizes frequency spectrum to distinguish mixed sources and calculate the corresponding phase of each spectrum peak.

**Figure 2:**
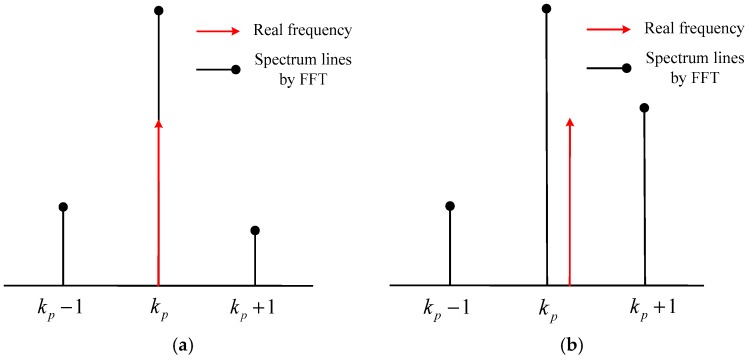
The relative position of the *p*th source frequency at the quantized frequency point: (**a**) Coincidence; (**b**) Mismatch.

The *m*th sensor’s frequency spectrum by employing Fourier Transformation algorithm can be expressed as

(12) $$X_{m}\left[k\right]=\sum_{n=1}^{N}x_{m}\left[n\right]\exp\left(-j2\pi \left(n-1\right)\left(k-1\right)/N\right)$$

for k=1,2,…,N, and the frequency resolution is $\Delta f=f_{s}/N$. The frequency estimation of the *p*th source can be obtained as

(13) $${\hat{f}}_{p}=f_{s}k_{p}/N$$

where $f_{s}$ is sampling frequency, $k_{p}$ is the position of the *p*th peak in the frequency spectrum. As shown in Figure 2a, the aforementioned method can estimate accurately when the source frequency is at the quantization frequency point. However, as shown in Figure 2b, there will be ±Δf/2 frequency estimation error if the source’s frequency lies between the two quantized points.

Therefore, the adjacent spectral lines of the peak are employed to improve the frequency estimation in this paper, which can be expressed as

(14) $$\hat{f_{p}}=\frac{f_{s}}{N}\left(k_{p}+g\frac{\left|X[k_{p}+g]\right|}{\left|X[k_{p}]|+|X[k_{p}+g]\right|}\right)$$

where g=−1 when $\left|X[k_{p}+1]|\leq |X[k_{p}-1]\right|$, g=1 when $\left|X[k_{p}+1]|\geq |X[k_{p}-1]\right|$. In this case, the error of frequency estimation is ±Δf/4, which is more accurate than employing only spectrum peaks to estimate frequency.

The phase difference of the *m*th centro-symmetric sensors for the *p*th source can be estimated from frequency spectrum and corresponding phase spectrum, which is free of range parameters and can be given by

(15) $$\begin{array}{ll} u_{m,p} & =\arg\left(X_{m}[k_{p}]\right)-\arg\left(X_{m+M/2}[k_{p}]\right)\\ & =(4\pi R{\hat{f}}_{p}/c)\cos(\gamma_{m}-\phi_{p})\sin\theta_{p} \end{array}$$

where $X_{m}[k_{p}]$ represents the value of the *p*th peak in the *m*th sensor’s frequency spectrum, $\arg(X_{m}[k_{p}])$ represents the phase of $X_{m}[k_{p}]$ in the exponent part, and ${\hat{f}}_{p}$ represents the frequency estimation of the *p*th source.

In order to avoid the phase ambiguity in $u_{m,p}$, the requirement R≤c/4f is assumed to guarantee $u_{m,p}\in [-\pi ,\pi )$. The phase ambiguity problem has already been well addressed in [14,15]. It can be noticed that $\cos(\gamma_{m}-\phi_{p})$ can be decomposed by employing angle transformation formula of trigonometric function, Therefore, by extending the scheme in [3] to decouple the phase difference $u_{m,p}$, we reformulate (15) as a form of two matrix multiplications

(16) U=AB

where

(17) $$U=\left[\begin{array}{cccc} u_{1,1} & u_{1,1} & \cdots & u_{1,P}\\ u_{2,1} & u_{2,2} & \cdots & u_{2,P}\\ \vdots & \vdots & \ddots & \vdots \\ u_{M/2,1} & u_{M/2,2} & \cdots & u_{M/2,P} \end{array}\right]$$

(18) $$A={\left[\begin{array}{cccc} \begin{array}{l} \cos(\gamma_{1})\\ \sin(\gamma_{1}) \end{array} & \begin{array}{l} \cos(\gamma_{2})\\ \sin(\gamma_{2}) \end{array} & \begin{array}{l} \cdots \\ \cdots \end{array} & \begin{array}{l} \cos(\gamma_{M/2})\\ \sin(\gamma_{M/2}) \end{array} \end{array}\right]}^{T}$$

(19) $$B=\frac{4\pi R}{c}\left[\begin{array}{c} \begin{array}{cccc} f_{1}\cos(\phi_{1})\sin(\theta_{1}) & f_{2}\cos(\phi_{2})\sin(\theta_{2}) & \cdots & f_{P}\cos(\phi_{P})\sin(\theta_{P}) \end{array}\\ \begin{array}{cccc} f_{1}\sin(\phi_{1})\sin(\theta_{1}) & f_{2}\sin(\phi_{2})\sin(\theta_{2}) & \cdots & f_{P}\sin(\phi_{P})\sin(\theta_{P}) \end{array} \end{array}\right]$$

for m=1,2,…,M/2 and p=1,2,…,P, and (•)^T^ denotes the transpose operator. It can be noticed that only the matrix B includes the unknown azimuth angles and elevation angles. By employing the least squares method, the optimal solution of B can be obtained as

(20) $$\hat{B}=\left[\begin{array}{c} \begin{array}{cccc} {\hat{b}}_{1,1} & {\hat{b}}_{1,2} & \cdots & {\hat{b}}_{1,P} \end{array}\\ \begin{array}{llll} {\hat{b}}_{2,1} & {\hat{b}}_{2,2} & \cdots & {\hat{b}}_{2,P} \end{array} \end{array}\right]={\left(A^{T}A\right)}^{-1}A^{T}U$$

where (•)^−1^ denotes the inverse operator. Therefore, on the basic of the *p*th vector of B, the *p*th source’s azimuth angle and elevation angle can be respectively obtained from (19)

(21) $${\hat{\phi }}_{p}=\arg({\hat{b}}_{1,p}+j{\hat{b}}_{2,p})$$

(22) $${\hat{\theta }}_{p}=\arcsin\left\{\left(c/4\pi Rf_{p}\right)\sqrt{{\hat{b}}_{1,p}^{2}+{\hat{b}}_{2,p}^{2}}\right\}$$

### 3.2. Range Estimation

It can be noticed in (9) and (10) that the array direction vector of the far-field source can be regarded as that of the near-field source in the case of infinity. Substituting the estimated azimuth angle and the elevation angle into the original array direction vector of the near-field source yields the direction vector of the *p*th source

(23) $$h\left({\hat{\phi }}_{p},{\hat{\theta }}_{p},r_{p}\right)={\left[h_{p,1},h_{p,2},\ldots ,h_{p,M}\right]}^{T}$$

with

(24) $$h_{p,m}=\exp\left\{\left(j2\pi Rf/c\right)\left[\zeta_{m}({\hat{\phi }}_{p},{\hat{\theta }}_{p})-\left(R/2r_{p}\right)\left(1-\zeta_{m}^{2}({\hat{\phi }}_{p},{\hat{\theta }}_{p})\right)\right]\right\}$$

According to the orthogonality of the signal subspace and the noise subspace, the function used for identifying mixed sources and estimating the range of the near-field source is constructed by employing 1-D multiple signal classification (MUSIC) method. Based on the estimated 2-D DOAs of each source, the range spatial spectrum of the *p*th source can be expressed as

(25) $$P_{r_{p},\mathrm{MUSIC}}\left({\hat{\phi }}_{p},{\hat{\theta }}_{p},r_{p}\right)={\left(h_{}^{*}\left({\hat{\phi }}_{p},{\hat{\theta }}_{p},r_{p}\right)VV_{}^{*}h\left({\hat{\phi }}_{p},{\hat{\theta }}_{p},r_{p}\right)\right)}^{-1}$$

where $V\in {\mathbb{C}}^{M\times (M-P)}$ represents the noise subspace and contains M−P eigenvectors which are corresponding to the related M−P small eigenvalues. The eigenvalues are obtained by employing eigendecomposition on the correlation function of received data. For finite sampling data, the correlation function can be estimated as $\left(1/N\right)XX^{*}$, where $X\in {\mathbb{C}}^{M\times N}$ represents the received data under UCA.

Ultimately, we classify far-field sources and near-field sources by searching the peak of range spatial spectrum. Just as frequency spectrum represents the energy distribution of the signal at each frequency, the range spatial spectrum is the energy distribution of the signal in space. The curves with obvious peak are corresponding to near-field sources, and the ranges of near-field sources can be estimated from the location of spectrum peaks. On the contrary, the curves of far-field source spatial spectrums are divergent.

**Figure 3:**
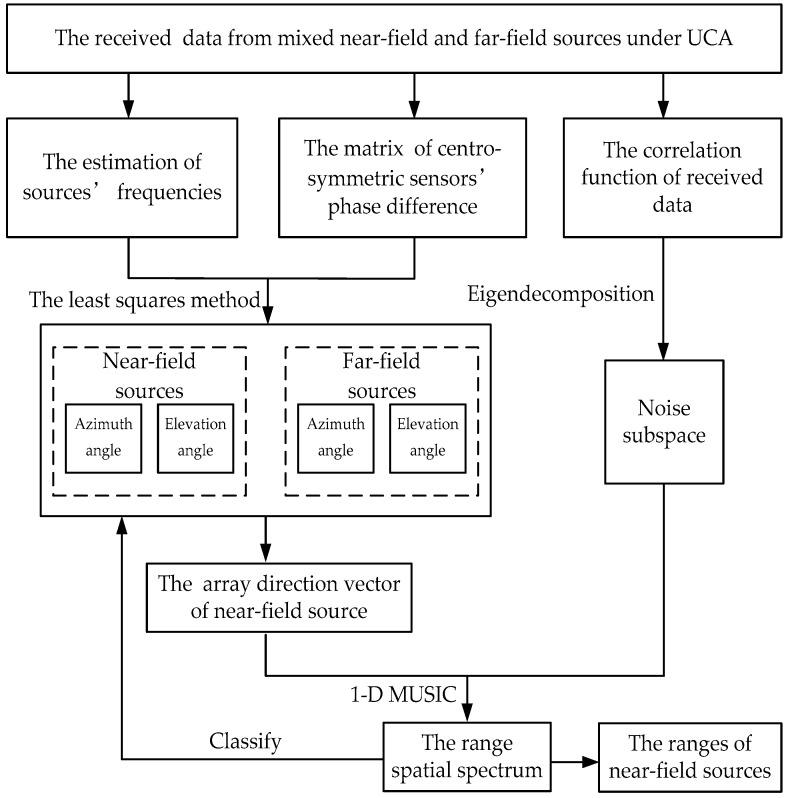
Flow chart of the proposed algorithm.

The flow chart of the proposed algorithm based on phase difference for mixed source localization is shown in Figure 3.

## 4. Simulation Results

In this section, some simulations are domonstrated to evaluate the effectiveness of our proposed algorithm. We utilize an eight-sensor UCA with radius R=0.6 m. Two equal power narrowband signals (one far-field source and one near-field source) are located at (47.1°, 37.2°) and (65.8°, 53.2°, 6 m) impinging on this array. The mixed source frequency and snapshot number are set at 100 MHz, 101 MHz and 3000, respectively. The additive noise is spatial white complex Gaussian random signal.

**Figure 4:**
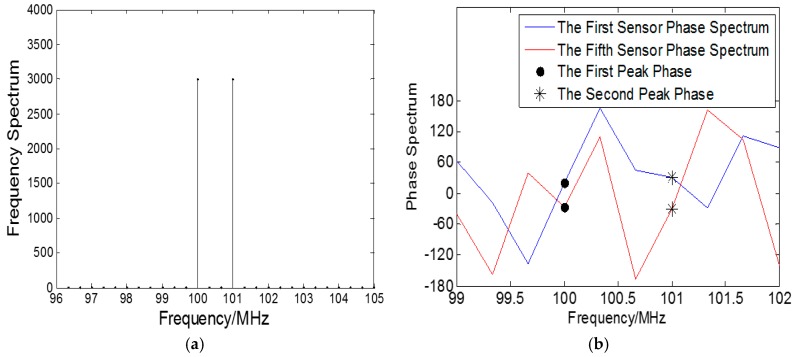
Mixed incoherent sources signal spectrums: (**a**) The first sensor frequency spectrum; (**b**) The first sensor and fifth sensors phase spectrum.

When signal-to-noise ratio (SNR) is set at 20 dB, the frequency spectrum of the first sensor by employing the Fast Fourier Transformation (FFT) algorithm is shown in Figure 4a, where black curve represents the frequency spectrum of the received data. It can be noticed that the incoherent sources can be resolved in frequency domain and the spectrum peaks are corresponding to the sources’ frequencies.

The phase spectrum of a set of centro-symmetric sensors are shown in Figure 4b, where the blue curve and the red curve represent the phase spectrum of the first sensor and the fifth sensor respectively. The black solid dots are the corresponding phases of the first spectrum peak in the frequency spectrum, and the black ∗ shape dots are the corresponding phases of the second spectrum peak in the frequency spectrum, which can be used to compute centro-symmetric sensors’ phase difference.

### 4.1. Performance of Range Estimation

**Figure 5:**
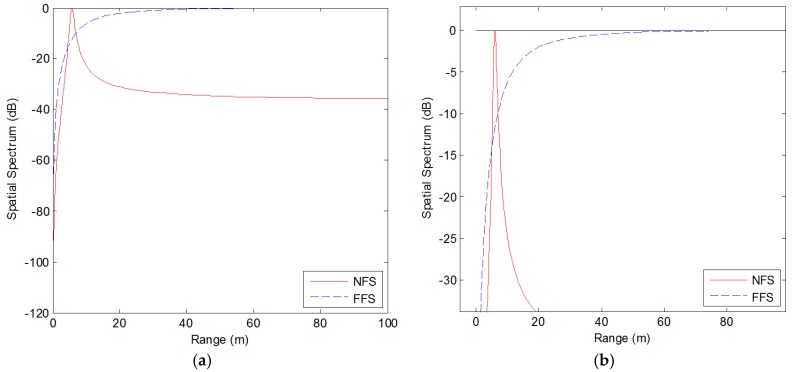
Spatial spectrum of range estimation: (**a**) Overall diagram; (**b**) Partial enlarged diagram.

In this simulation, we verify the range estimation and recognition performance of our proposed algorithm when SNR is set at 20 dB. The range spatial spectrum of far-field source and near-field source are shown in Figure 5, where the solid curve in red color represents the range spatial spectrum of near-field source, and the dashed curve in blue color represents that of far-field source. It is noticed that there is a peak in the range spatial spectrum, which is corresponding to the range of the near-field source. Furthermore, the mixed sources can be identified from the range spaital spectrum successfully. The curve of the near-field source in red color is convergent, which has an obvious peak. On the contrary, the curve of the far-field source in blue color is divergent. It can be seen from the enlarged diagram in Figure 5b that the blue curve is close to 0 dB and there is no tiny little peak in the range spatial spectrum.

### 4.2. Performance of Location

**Figure 6:**
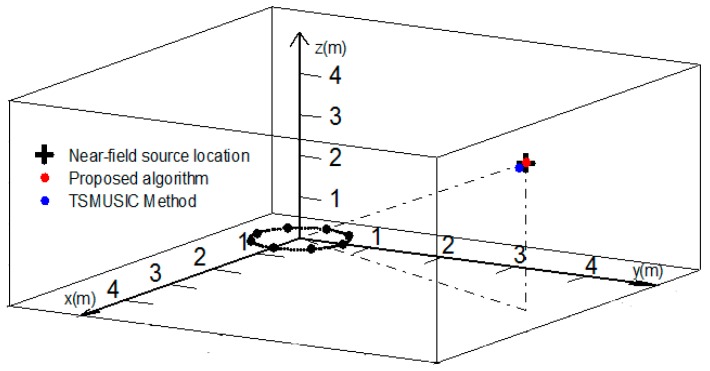
Positioning result of the near-field source (SNR = 20 dB).

In this section, in order to further demonstrate the superiority of the proposed method for mixed source localization, we compare the TSMUSIC method in [13] to make the performance comparison when SNR is set at 20 dB. The state-of-the-art first employed differencing matrix to separate near-field sources from mixed sources and obtain the 2-D DOAs of near-field sources. Then, 1-D MUSIC method and 2-D MUSIC method are utilized to obtain the ranges of near-field sources and the 2-D DOAs of far-field sources, respectively. The position simulation and comparisons are shown in Table 1 and Figure 6. As the spacing of the array sensors used to calculate the phase difference increases, the accuracy of parameter estimation by employing phase-based method becomes higher and higher [14]. Accordingly, due to the fact that the spacing of centro-symmetric sensors used in this paper is the longest under UCA, it can be seen that the proposed algorithm for the angle estimation has higher accuracy than the TSMUSIC algorithm with step size = 0.01°. In addition, due to the fact that 1-D MUSIC method is based on the 2-D DOAs to estimate the near-field source’s range, and the estimated 2-D DOAs by utilizing the phase-based method are more accurate, the performance of the proposed method is better than TSMUSIC method with step size = 0.01 m for the near-field source’s range estimation.

As shown in Figure 6, the + shape represents the real location of the near-field source, the red dot represents the location of the near-field source by employing the proposed method, and the blue dot represent the location of the near-field source by employing TSMUSIC method. It is can be noticed that the proposed algorithm and the TSMUSIC method can achieve the localization of the near-field source. Moreover, the positioning result of the proposed algorithm is more precise than that of the TSMUSIC method.

**Figure 7:**
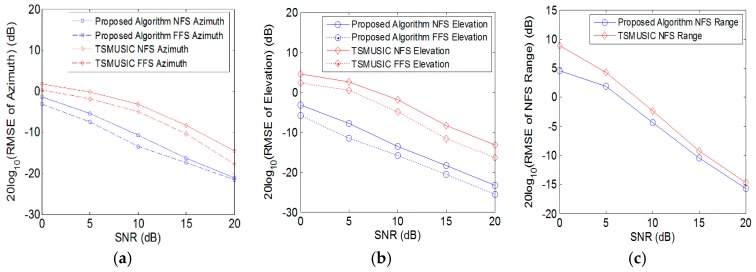
RMSEs versus SNR: (**a**) Azimuth angle; (**b**) Elevation angle; (**c**) Near-field source range.

In order to further demonstrate the superior estimation performance of the proposed method for mixed source localization, we compare the root mean square errors (RMSEs) of the proposed algorithm to those of the TSMUSIC method in [13], and the results are obtained from 500 independent Monte Carlo simulations. The logarithm of the RMSEs of the azimuth angle, elevation angle and the near-field source’s range are given in Figure 7, where the solid curves in blue color represent the RMSEs of the near-field source by employing the proposed method, the dashed curves in blue color represent the RMSEs of the far-field source by employing the proposed method, the solid curves in red color represent the RMSEs of the near-field source by employing TSMUSIC method, and the dashed curves in red color represent the RMSEs of the far-field source by employing TSMUSIC method, respectively. It can be noticed that the proposed algorithm for the angle estimation has higher accuracy than TSMUSIC algorithm with step size = 0.01°. 

Both the proposed method and the TSMUSIC method employ the 1-D MUSIC to obtain the ranges of the near-field sources. However, the 1-D MUSIC method is based on the 2-D DOAs to estimate the near-field source’s range, and the estimated 2-D DOAs by utilizing the proposed method are more accurate, it can be noticed that the performance of proposed method is better than TSMUSIC method with step size = 0.01 m for the near-field source’s range estimation.

As for the computational cost, compared with the TSMUSIC algorithm with step size = 0.01° for angle estimation and step size = 0.01 m for range estimation, due to the fact that TSMUSIC algorithm requires an expensive search process, the elapsed CPU time for a single run of our proposed method and TSMUSIC algorithm are measured as 0.26 s and 50.95 s, respectively. It is noteworthy that the proposed method has lower computational complexity.

### 4.3. Performance of Location under Frequency Estimation Error

In this section, considering that the frequency calculated by FFT algorithm may not correspond to the frequency of the source. The RMSEs obtained from 500 independent Monte Carlo simulations are employed to show the effect of frequency estimation error. 

**Figure 8:**
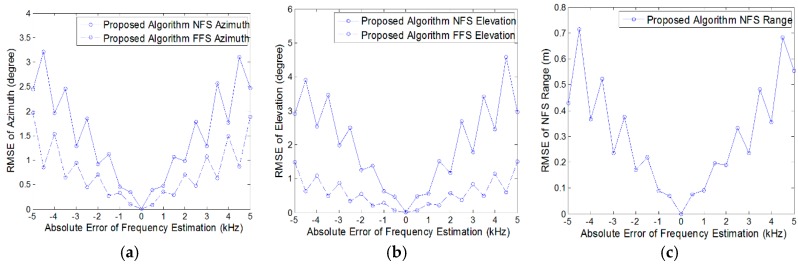
RMSEs versus absolute error of frequency estimation (SNR = 20 dB): (**a**) Azimuth angle; (**b**) Elevation angle; (**c**) Near-field source range.

When the SNR is set at 20 dB, we assume that the error between the estimated frequency and the actual frequency is a priori information, the RMSEs of the azimuth angle, elevation angle and the near-field source’s range are given in Figure 8, where the solid curves in blue color represent the RMSEs of the near-field source and the dashed curves in blue color represent the RMSEs of the far-field source respectively. It can be noticed that the RMSEs of 3-D position parameters are showing an increasing trend with the increase of frequency absolute error. Moreover, compared with the RMSEs of near-field source’s angles, the RMSEs of far-field source’s angles are relatively small. The RMSEs of angles are approximately within 1° when the frequency absolute error is within 2 kHz, and the RMSEs of range are approximately within 0.2 m when the frequency absolute error is within 2 kHz.

## 5. Conclusions

This paper has presented a phase-based method for estimating the azimuth angles and elevation angles of the mixed incoherent sources under UCA. We first decouple angle parameters from array direction vector by calculating centro-symmetric sensors’ phase differences. After that, as the phase differences only include sources’ angle parameters and can be transformed as indefinite equations, each source’s angles are obtained by performing the least squares method. Meanwhile, substituting the obtained 2-D DOAs into the original steering vector of near-field source, this paper employs 1-D MUSIC method to distinguish the mixed sources and obtain the ranges of near-field sources.

The advantages of our proposed algorithm are that it can obtain the 2-D DOAs of each source simultaneously and identify mixed sources by employing range spatial spectrum. Considering that the localization performance of the proposed method is related to the accuracy of the frequency estimation, we employ the adjacent spectral lines of the spectrum peak to improve frequency estimation. Compared with TSMUSIC method by employing the estimated root mean square errors, the proposed method has higher estimation accuracy with lower computational cost. In addition, the proposed algorithm can also be applied in the case of the multiple near-field sources or multiple far-field sources localization.

## Figures and Tables

**Table 1:** Mixed source parameter estimation comparison (SNR = 20 dB).

Parameter Estimation	Actual Parameter		Proposed Method		TSMUSIC Method ^1^	
	NFS ^2^	FFS ^3^	NFS	FFS	NFS	FFS
Azimuth angle (degree)	65.8	47.1	65.82	47.10	66.05	46.81
Elevation angle (degree)	53.2	37.2	53.18	37.20	53.89	37.39
Range (m)	6	∞	6.00	∞	5.91	∞

^1^ represents the method in [13]; ^2^ represents the near-field source; ^3^ represents the far-field source.
